# The Associations between Cytokine Levels, Kidney and Heart Function Biomarkers, and Expression Levels of Angiotensin-Converting Enzyme-2 and Neuropilin-1 in COVID-19 Patients

**DOI:** 10.3390/vaccines10071045

**Published:** 2022-06-29

**Authors:** Rabab Hussain Sultan, Maged Abdallah, Tarek M. Ali, Amr E. Ahmed, Hebatallah Hany Assal, Basem H. Elesawy, Osama M. Ahmed

**Affiliations:** 1 Biotechnology and Life Sciences Department, Faculty of Postgraduate Studies for Advanced Sciences, Beni-Suef University, Beni-Suef 62521, Egypt; amreahmed@psas.bsu.edu.eg; 2 Department of Anesthesia and Intensive Care, Faculty of Medicine, Cairo University, Cairo 11562, Egypt; maged.salah@kasralainy.edu.eg; 3 Department of Physiology, College of Medicine, Taif University, P.O. Box 11099, Taif 21944, Saudi Arabia; tarek70ali@gmail.com; 4 Department of Chest Medicine, Faculty of Medicine, Cairo University, Cairo 11562, Egypt; hebatallah.assal@kasralainy.edu.eg; 5 Department of Pathology, College of Medicine, Taif University, P.O. Box 11099, Taif 21944, Saudi Arabia; basemelesawy2@gmail.com; 6 Physiology Division, Zoology Department, Faculty of Science, Beni-Suef University, P.O. Box 62521, Beni-Suef 62521, Egypt; osama.ahmed@science.bsu.edu.eg

**Keywords:** COVID-19, ACE-2 and NRP-1 expression, cytokines, cardiac enzymes, kidney function

## Abstract

Background: Higher expression of angiotensin-converting enzyme-2 (ACE-2) in addition to neuropilin-1 (NRP-1) can lead to a cytokine storm which is correlated to higher mortality rate and contributes to the progression of renal diseases and the pathogenesis of coronary heart disease (CHD) in COVID-19 patients. Aim: We herein sought to examine correlations between cytokine levels, ACE-2 and NRP-1 expression, renal function biomarkers, and cardiac enzymes in COVID-19 patients. Patients and Methods: For the study, 50 healthy subjects and 100 COVID-19 patients were enrolled. Then, confirmed cases of COVID-19 were divided into two groups—those with moderate infection and those with severe infection—and compared to healthy controls. Serum creatinine, urea, CK-MB, LDH, troponin I, IL-1β, IL-4, IL-10, IL-17, and INF-γ levels were estimated. We also studied the gene expression for ACE-2 and NRP-1 in blood samples utilizing quantitative real-time polymerase chain reaction (qRT-PCR). Results: All COVID-19 patients demonstrated a significant increase in the levels of serum creatinine, urea, CK-MB, LDH, and troponin I, as well as examined cytokines compared to the healthy controls. Furthermore, ACE-2 mRNA and NRP-1 mRNA expression levels demonstrated a significant increase in both severe and moderate COVID-19 patient groups. In the severe group, serum creatinine and urea levels were positively correlated with IL-10, INF-γ, NRP-1, and ACE-2 expression levels. Moreover, LDH was positively correlated with all the examined cytokine, NRP-1, and ACE-2 expression levels. Conclusion: Deficits in renal and cardiac functions might be attributable to cytokine storm resulting from the higher expression of ACE-2 and NRP-1 in cases of COVID-19.

## 1. Introduction

Severe acute respiratory syndrome coronavirus 2 (SARS-CoV-2), a recent coronavirus form, first appeared in Wuhan, China in December 2019 and the COVID-19 pandemic was declared in March 2020. By this time, COVID-19 had been described in more than 220 countries with around 118 million established cases and nearly three million deaths [1]. Clinically, this disease ranges from cases with no symptoms to cases with severe respiratory symptoms or respiratory failure, sepsis of multiple systems, septic shock, and impairment of various organ [2].

The way in which the innate immune system can respond to viral infection is shown by the facts available on interventions in viral actions, metabolic dysfunctions, and cytokine-triggering molecular mechanisms [3,4,5]. Multiple theories suggest patients with severe COVID-19 can present with a cytokine storm syndrome (CSS), which has an important effect in severe cases of COVID-19 pathophysiology and acute respiratory distress syndrome (ARDS). Inflammatory processes mediated by cytokines have been connected with the pathogenesis of kidney insult either by acute kidney injury (AKI) or by chronic kidney disease (CKD) [6]. Chronic inflammation characterized by high levels of pro-inflammatory and inflammatory cytokines is strongly connected with coronary heart disease, heart attacks, cardiac failure, and further harmful cardiac events. Cytokines, through induction of the inflammatory cascading, contribute to the progress of atherosclerotic processes and enhance multiple facets of atherogenesis [7]. Interleukins, chemokines, interferons, and tumor necrosis factors are all small cytokines and nonstructural proteins that have several pleiotropic effects in many organs [1]. In many infections and disorders of the immune system, they are released by paracrine, autocrine, or endocrine pathways and exhibit pro-inflammatory or anti-inflammatory processes. Cytokines that have pro-inflammatory reactions include interleukin (IL)-1β (IL-1β), IL-17, interferon-γ (IFN-γ), and tumor necrosis factor-*α* (TNF-*α*) [2,6], Those with anti-inflammatory properties include interleukins (ILs)-10 and -4. The recent identification of the first route of viral entry was mediated by the angiotensin-converting enzyme-2 (ACE-2), which is represented in different human organs such as the kidneys, heart, lungs, small intestine, thyroid, testes, and adipose tissue [8]. ACE-2 catalyzes the conversion of angiotensin-(1–8) to angiotensin-(1–7), then the ACE-2/angiotensin-(1–7)/MAS axis opposes the adverse properties of the renin–angiotensin system (RAS) that performs crucial functions in preventing the functional and pathogenic imbalance of the human body [8]. Reports stated that SARS-CoV infection can lower cellular ACE-2 expression and raise the levels of blood, urine, and other body fluids soluble ACE-2 [9]. ACE-2 down-regulation, and the RAS and ACE-2/angiotensin-(1–7) disproportion following infection can impact several organs in COVID-19 patients [8]. The cell entry of coronaviruses is determined by the attachment of the viral spike (S) proteins into cellular receptors. S proteins are primed by host cell proteases such as the transmembrane serine protease 2 [10]. SARS-CoV-2 spike (S) protein has more than a 10-fold greater affinity to bind ACE-2 than SARS-1 S protein [11]. To date, attention has been entirely focused on ACE-2 as a receptor, which is a likely objective for emerging specialized treatments, antibodies, and vaccines. The restoration of the equilibrium between the RAS and ACE-2 can help reduce organ-related injuries. Recent research has identified the significance of neuropilin-1 (NRP-1) as a new type of cell surface receptor in the virus’s entry to the host cells and the subsequent viral multiplication. SARS-CoV-2, in comparison to SARS-1, is more communicable and persistent. Its rapid dissemination via active shedding of the pharyngeal mucosa, the availability of a furin-type cleavage site at its spike protein, and the usage of the two receptors ACE-2 and NRP-1 in order to infect cells could all be factors [12]. NRP-1 is controlled by the mammalian target of the rapamycin pathway. A stimulated NRP-1 receptor can augment the response of the dendritic cells (DCs) of the immune system toward SARS-CoV-2 to encourage the cytokine storm, the main cause of the high mortality rate in COVID-19 [11,13]. Acute renal failure continues to be of concern in patients suffering COVID-19. Several factors are responsible for the etiology of kidney impairment in COVID-19 together with the significant role of the inordinate cytokine release [14].

New onset of heart failure (HF) has been detected in about 25% of hospitalized COVID-19 patients and in ~33% of the intensive care unit (ICU) patients regardless of the presence of HF history [15,16]. In addition, patients who survive COVID-19 have a higher risk of kidney complications in the post-acute phase of the illness [17]. Moreover 30% of COVID-19 patients who are hospitalized experience kidney impairment, according to a previous publication [18]. Furthermore, compared to the general population, patients with end-stage kidney disease, particularly those undergoing in-center hemodialysis, may have a higher risk of infection and mortality [19]. Thus, the current study aimed to examine the relationships between serum cytokine levels, serum kidney function biomarkers, and cardiac enzymes, and the expression of ACE-2 and NRP-1 receptors in COVID-19 patients.

## 2. Patients and Methods

### 2.1. Study Population

A cohort of 100 volunteers confirmed to have been COVID-19 patients (mean age 57.74 years) was included in this study. All participants were isolated at Misr International Hospital, Cairo, Egypt, in the period from March 2021 to April 2021. The research protocol of the present study was conducted following the Helsinki Declaration and guidelines for good practice. The study was approved by the ethics committee of Misr International Hospital, Cairo, Egypt and the institutional review board of the Ministry of Health, Cairo, Egypt (No: 3 – 2021/19). The selected patients were considered to be COVID-19 patients as their chest CT images had the characteristic features suggestive of COVID-19 lung infection. The characteristics of these CT images comprised lung opacity (ground-glass, consolidative, or crazy-paving patterns), septal thickening, and the reverse halo sign [20]. Reverse transcription-polymerase chain reaction (RT-PCR) was the reference benchmark for laboratory testing of SARS-CoV-2 [21]. All patients included in the study had an RT-PCR-positive COVID-19 test.

### 2.2. Patients

This study included 150 people (both men and women): 50 healthy people and 100 COVID-19 sufferers. Based on how severe their symptoms were, confirmed cases of COVID-19 were divided into two groups [22]. Patients with symptoms such as fever and respiratory tract symptoms and pneumonia manifestation that could be seen in imaging were considered moderate cases. Patients with respiratory distress (respiratory rate ≥ 30 breaths/min, SpO2 ≤ 93% at rest, and PaO2 /FIO2 ≤ 300) and patients with greater than 50% lesion progression within 24 to 48 h in pulmonary imaging were considered severe cases [22].

The participants were placed into three groups:

**Group I:** Healthy controls (n = 50, 25 men and 25 women).

**Group 2:** COVID-19 patients with moderate disease (n = 50, 25 men and 25 women).

**Group 3:** COVID-19 patients with severe symptoms (n = 50, 29 men and 21 women).

### 2.3. Inclusion and Exclusion Criteria

Healthy controls were COVID-19 free. All patients had COVID-19 identified by PCR and chest CT, according to the World Health Organization (WHO) 2020. Participants were aged between 18 and 70. Primary exclusion criteria included hypertensive patients treated with ACE-2 inhibitors as well as patients with pre-existing thyroid dysfunction, autoimmune disorders, respiratory disorder, kidney or liver failure, cerebrovascular diseases, ischemic heart disease, and pregnant and lactating women. Patients taking immune-suppressing medicines or suffering from medical illnesses such as infections, cancer, or alcoholism were also eligible for exclusion.

### 2.4. Demographic Data

Demographic data in terms of anthropometric variables such as height, weight, body mass index (BMI), and the gender were gathered.

### 2.5. Blood Samples

Participants’ blood samples were collected in plain tubes (4 mL each). Blood in plain tubes was centrifuged for serum isolation after a 30 min incubation period at room temperature. Serum samples were quickly separated, aliquoted, and maintained at −40 °C until measurements were conducted. The second tube contained a blood sample that was taken on potassium fluoride for quick glucose analysis. 

The levels of ACE-2 receptor mRNA expression and NRP-1 receptor mRNA expression in blood samples were examined in the healthy-control, moderate, and severe groups [23] using quantitative real-time PCR (qRT-PCR). Procedures of gene sequence (5′-3′) were conducted as per the kit instructions included in the laboratory assay.

### 2.6. Laboratory Assays

Serum creatinine concentration was determined as per Henry’s approach (1974) [23] using reagent kits obtained from Diamond Diagnostics (Egypt). Blood urea concentration was determined as per Kaplan’s approach (1984) [24] using a reagent kit from Diamond Diagnostics (Cairo, Egypt). Lactate dehydrogenase (LDH) activity in serum was assessed as per Buhl and Jackson’s approach (1978) [25] using reagent kits from Stanbio Laboratories (Boerne, TX, USA).

To measure serum CK-MB, troponin I, and ILs-1, -4, -10, and -17, as well as INF, researchers used a standard sandwich enzyme-linked immunosorbent assay kit from Innovative Systems (USA) and followed manufacturer’s instructions.

### 2.7. RNA Isolation and qRT-PCR

Total RNA was isolated from blood samples of individuals in each group by a method that used TRIzol reagent (MBI Fermentas, Germany), and cDNA was generated as per the manufacturer’s instructions using the high-capacity cDNA Reverse Transcription Kit (Invitrogen, Germany). A 20 L apparatus with 10 liters of 1x Sso-Fast Eva Green Supermix was used for real-time PCR (Bio-Rad, Hercules, CA, USA), 2 μL of cDNA, 6 μL of RNase/DNase-free water, and 500 nM of the primer pair sequences:

NRP-1, F-5′ AACAACGGCTCGGACTGGAAGA 3′ and R-5 GGTAGATCC

TGATGAATCGCGTG 3′ (NM001024628). ACE-2, F-5′ TCCATTGGTCTTC

TGTCACCCG 3′ and R-5′ AGACCATCCACCT CCACTTCTC 3′ (NM021

804.3), β–actin, F-5′ GGAACGGTGAAGGTGACAGCAG 3′ and R-5′ TGT

GGACTTGGGAGAGGACTGG 3′(XM004268956.3).

The thermal cycler requirements were as follows: 30 s at 95 °C, and then 40 cycles of 5 s at 95 °C and 10 s at 60 °C. For every one reaction, a 65–95 °C ramp was conducted with a melting curve study. Using each procedure, the threshold duration at which the fluorescent signal had gone beyond an indiscriminately defined threshold, such as the middle of the log-linear amplification step, was assessed, and the relative quantity of mRNA was determined. Amplified data were analyzed by the manufacturer’s program using Livak and Schmittgen’s approach [26], Following that, the variables were standardized to β-actin.

### 2.8. Statistical Analysis

Values are displayed as means ± standard errors of means (SEM). Statistical Package for the Social Sciences (SPSS) version 22 for Windows (New York, USA) was used for analyze the data [27]. Duncan’s post-hoc test was applied to compare the groups. Pearson’s correlation coefficient analysis was used to estimate correlation among different study parameters. Values of *p* < 0.05, *p* < 0.01, and *p* < 0.001 were statistically significant at three levels whereas values of *p* > 0.05 were statistically non-significant.

## 3. Results

Table 1 shows demographic data of the studied groups. Group 1 is the healthy control group that included 50 heathy volunteers (25 males and 25 females) aged 52.12 ± 1.22 years with BMI of 24.25 ± 0.35 kg/m^2^. Group 2 included 50 patients (25 males and 25 females) with moderate COVID-19 aged 58.24 ± 1.24 with BMI of 24.73 ± 0.48 kg/m^2^. Group 3 included 50 patients (29 males and 21 females) with severe COVID-19 aged 57.36 ± 1.43 with BMI of 25.03 ± 0.52 kg/m^2^.

**Figure 1:**
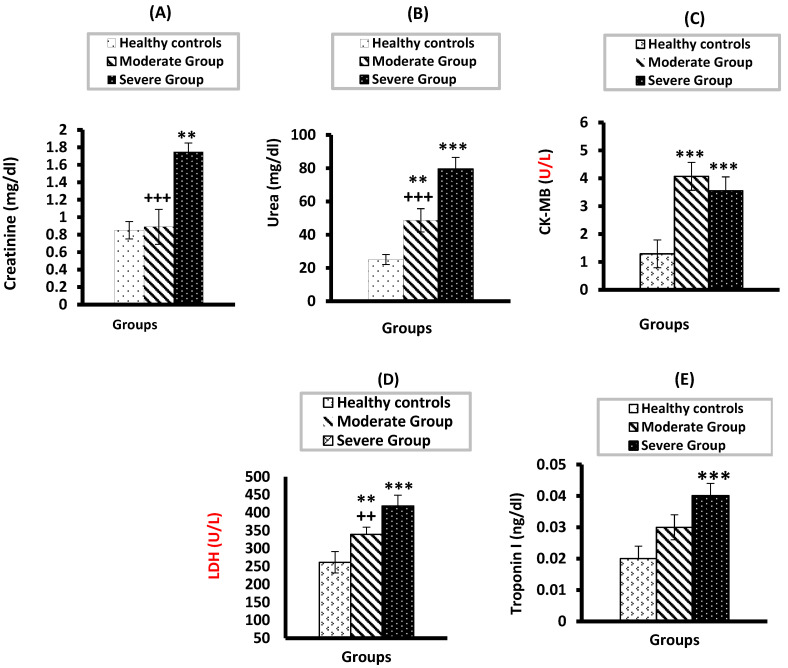
Serum levels of creatinine (**A**), urea (**B**), CK-MB (**C**), LDH (**D**), and Troponin (**E**) in moderate and severe COVID-19 groups compared to healthy control. ** and *** significant compared to heathy control at *p* < 0.01 and *p* < 0.001, respectively; ^+++^ significant compared to severe group at *p* < 0.001; ^++^ significant compared to severe group at *p* < 0.01.

Figure 1 shows the levels of the kidney and heart function biomarkers in serum of all groups. Relative to the healthy control and moderate group, the severe group’s serum levels of creatinine (Figure 1A) and urea (Figure 1B) were significantly higher. Serum creatinine and urea levels showed a significant rise in the severe COVID-19 patients compared with the moderate group (*p* < 0.001). It was also found that the activities of serum CK-MB (Figure 1C) and LDH (Figure 1D) were significantly higher in the two COVID-19 groups versus the healthy group. There was no difference (*p* > 0.05) in CK-MB activity in the moderate versus the severe COVID-19 group. However, LDH activity demonstrated a significant (*p* < 0.01) increase in the severe group versus the moderate group. Troponin I demonstrated a significant rise only in the severe group (*p* < 0.01) in comparison to the control group with no significant difference found between the two COVID-19 groups (Figure 1E).

**Figure 2:**
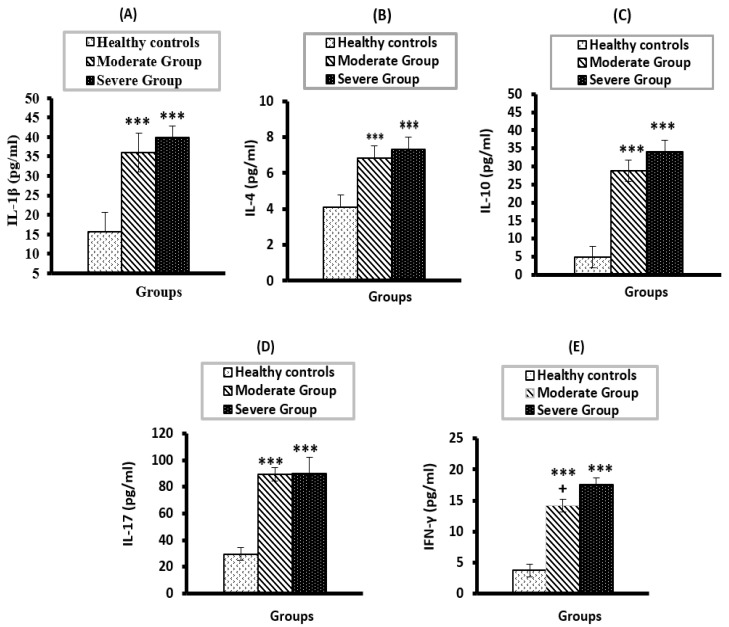
Serum levels of IL-1β (**A**), IL-4 (**B**), IL-10 (**C**), IL-17 (**D**), IFN-γ (**E**) in moderate and severe COVID-19 groups compared to healthy control. *** significant compared to heathy control at *p* < 0.001; ^+^ significant compared to severe group at *p* < 0.05.

Both COVID-19 groups demonstrated a significant elevation (*p* < 0.001) in IL-1β, IL-4, IL-10, IL-17, and IFN-γ as compared to healthy controls (Figure 2A–E). No significant differences were observed between the two COVID-19 groups except for INF- γ (*p* < 0.05).

**Figure 3:**
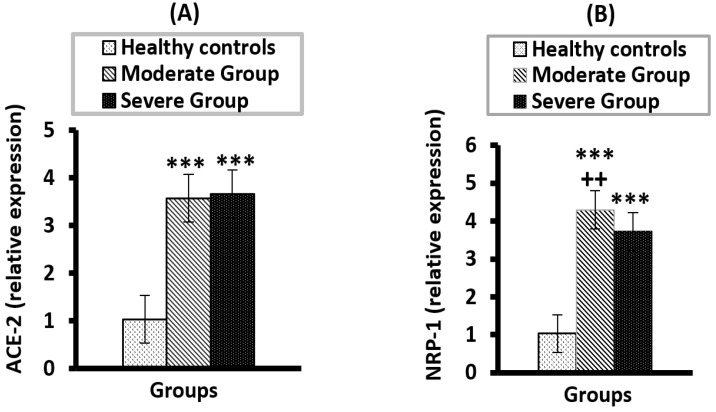
The mRNA expression of ACE-2 (**A**) and NRP-1 (**B**) in moderate and severe COVID-19 groups compared to healthy control. *** significant compared to heathy control at *p* < 0.001; ^++^ significant compared to severe group at *p* < 0.01.

ACE-2 and NRP-1 mRNA expression are represented in Figure 3A,B respectively. Both exhibited a considerable increase (*p* < 0.001) in the moderate and severe groups versus the healthy group (Figure 3A,B) with no significant (*p* > 0.05) differences observed when we compared the two diseased groups in terms of ACE-2 expression. However, when compared to the severe group, NRP-1 expression levels were considerably greater (*p* < 0.01) in the moderate group.

Table 2 shows the correlation between the kidney and heart function biomarkers, and cytokine, ACE-2, and NRP-1 expression levels in the moderate group. In the moderate COVID-19 patient group, serum creatinine demonstrated a significant (*p* = 0.000, *p* = 0.029) positive association only with serum urea and LDH. A considerably negative association with IL-1β (*p* = 0.036), but a slight negative correlation between serum creatinine and IL-4, IL-10, IL-17, IFN-γ, NRP-1, and ACE-2 expression (*p* > 0.05) were observed. A positive insignificant (*p* > 0.05) correlation was reported between serum creatinine and CK-MB and troponin I. However, blood urea demonstrated a substantial (*p* < 0.05) positive association with creatinine, LDH, CK-MB, IL-1β, IL-4, IL-17, and NRP-1 expression levels. There was an insignificant positive (*p* > 0.05) correlation between serum urea and troponin I, IL-10, and ACE-2 expression levels. 

CK-MB demonstrated a positive significant (*p* < 0.05) correlation with urea, troponin I, IL-1β, IL-4, IL-10, IL-17, IFN-γ, NRP-1, and ACE-2 expression levels. Insignificant (*p* > 0.05) correlation was reported between CK-MB and creatinine levels (positive). LDH demonstrated a positive significant (*p* < 0.05) correlation with creatinine, urea, IL-4, IL17 IFN-γ, and NRP-1 expression levels. However, LDH demonstrated a positive insignificant (*p* > 0.05) correlation with CK-MB, troponin I, IL4, IL10, IFN-γ, and ACE-2 expression levels. There was a positive significant (*p* < 0.05) link between age and troponin I and CK-MB, IL-4, IL-10, IL-17, ACE-2, and NRP-1 expression levels. Troponin I demonstrated a positive insignificant (*p* > 0.05) correlation with creatinine, urea, LDH, IL-1β, and IFN-γ.

Table 3 shows the correlation between the kidney and heart function biomarkers and cytokine, ACE-2, and NRP-1 expression levels in the severe group. Serum creatinine demonstrated a considerably (*p* < 0.05) positive association with urea, CK-MB, IL-10, IFN-γ, NRP-1, and ACE-2 expression levels. However, there was an insignificant negative (*p* > 0.05) correlation between serum creatinine and troponin I. Furthermore, there was an insignificant positive correlation with LDH, IL-1β, IL-4, and IL-17.

However, serum urea demonstrated a substantial (*p* < 0.05) positive relationship with creatinine, LDH, CK-MB, IL-1β, IL-4, IL-10, IL-17, IFN-γ, and ACE-2 and NRP-1 expression levels. There was an insignificant negative (*p* > 0.05) correlation between urea and troponin I. 

CK-MB demonstrated a positive significant (*p* < 0.05) correlation with creatinine, urea, LDH, troponin I, IL-1β, IL-4, IL-10, IL-17, IFN-γ, NRP-1, and ACE-2 expression levels. 

LDH demonstrated a positive significant (*p* < 0.05) correlation with urea, troponin I, IL-1β, IL-4, IL-10, IL-17, IFN-γ, NRP-1, and ACE-2 expression levels. There was a positive significant (*p* < 0.05) association between troponin I and LDH, CK-MB, IL-4, IL-17, and NRP-1 expression levels. However, troponin I demonstrated a positive insignificant correlation with IL-1β, IL10, IFN-γ, and ACE-2 expression levels. It also demonstrated an insignificant (*p* > 0.05) correlation with creatinine, and urea.

## 4. Discussion

COVID-19′s epidemiological and clinical characteristics and its effects on the levels of both types of cytokines (pro- and anti-inflammatory), kidney and heart function biomarkers, and ACE-2 and NRP-1 expression levels are presented in this study.

This novel coronavirus, SARS-CoV-2 is known to induce cytokine storm syndrome (CSS) [28]. The CSS is frequently described as causing high death rates. Cron and Behrens reported CSS as a storm that activates a cascade of auto-augmenting cytokine manufacturing because of a disordered host immune response to various causes such as infections or cancer [29]. Guan et al. demonstrated a significant leukocytosis, neutrophilia, and higher levels of procalcitonin, C-reactive protein, and various other inflammatory indicators in the ICU COVID-19 patients, versus non-ICU-admitted patients [30].

Xu et al. discovered the presence of ARDS as well as T-cell over-activation. This effect was attributed to the increased number of T-helper (Th) 17 cells as well as the treble cytotoxicity of CD8^+^ T cells [31]. The uninhibited CSS and inflammatory responses induced by SARS-CoV-2 help activate the immune response—either innate or adaptive reactions [32]. In both moderate and severe COVID-19 groups, we reported an increase in cytokines such as ILs-1β, -4, -10, and -17, as well as IFN-γ. IL-1 is a pro-inflammatory cytokine involved in inflammatory disorders such as infections and autoimmune disease. It is divided into two types: IL-1α and IL-1β [33]. It is primarily produced by macrophages and other tissues and synthesized by tubular, endothelial, mesangial, epithelial, and renal cells. IL-1 interconnects with all the other cytokines and can proceed as either autocrine or paracrine to produce nephrotoxicity, and is thus implicated in several renal diseases [34,35]. The significant increase in IL-10 might be regarded as trying to control excessive inflammation and minimize tissue-related harm, based on its abilities as an anti-inflammatory and immunosuppressant cytokine. Furthermore, the association seen between higher IL-10 levels and the severity of the disease, as well as synchronized increases in IL-10 and several pro-inflammatory cytokines, suggest that IL-10 fails to suppress inflammation or acts in a different way to that of its known role as an anti-inflammatory cytokine [36].

Possible explanations and implications of the raised IL-10 levels in sufferers from COVID-19 can be discussed in the following manner. First, intense activation of the CD8^+^ T cells by high IL-10 levels gives rise to T-cell overactivity and strengthens IFN-γ levels. IFN-γ stimulates the production of pro-inflammatory factors using activated macrophages. Second, IL-10 “resistance” reduces stimulated monocyte/macrophage response to flowing IL-10, thus boosting the release of inflammatory cytokines [36]. Through up-regulating inhibitors of some cytokines and cleaning the receptors, IL-4 displays its anti-inflammatory character [37]. IL-4 cells are formed from Th2 cells, which reportedly present in considerable quantities in COVID-19 sufferers [38]. By self-activating receptors on Th1 cells, IL-4 is the main cause of the immune system’s change from Th1 to Th2 [39]. Th17 cells create IL-17 to engage monocytes, macrophages, and neutrophils and stimulate cytokines, comprising IL-1β, and IL-6 [40,41]. IL-17 is a pro-inflammatory cytokine and plays a pivotal role in both host defense mechanisms against infection and in immune regulation [42]. In accordance with the results of the present study, Martonik et al. [43] reported that patients with severe COVID-19 exhibit high levels of IL-17. In addition, Martonik et al. [43] suggested that lung tissue destruction could be explained by neutrophil recruitment mediated by Th17 cells. In the present study, serum IL-17 was observed to have a positive connection with serum urea, CK-MB, LDH, and troponin I in both moderate and severe COVID-19 patients. Thus, the elevation in IL-17 may be linked with the deteriorations in kidney and heart functions in addition to its roles in lung tissue destruction.

CD4^+^ T cells are a critical component of the immune system that orchestrates adaptive immune responses. Naive CD4 T cells are triggered when antigen-presenting cells, for example in the DCs, deliver pathogen signals to CD4^+^ T cells, which then differentiate to separate T effector (T_eff_) populations according to the variety of infection. By producing active cytokines, effector CD4^+^ T cells offer host defense mechanisms against pathogens, which turn on other immune cells such as macrophages and CD8^+^ T cells, destroying infected cells and helping B cells to produce the antibodies mediating humoral immune responses [44]. Major CD4^+^ T helper cell (Th) subsets, such as Th1, Th2, and Th17, have been recognized. Th1 cells are known by their ability to express IFN-γ, contributing to type 1 immune response to intracellular pathogens such as viruses. Shao et al. reported that infusion of angiotensin II caused a Th1-type reaction and renal damage in the hypertension rat model [45]. Th2 cells can express ILs-4, -5, and -13 and engage in type 2 immune response against larger extracellular pathogens such as helminths. In the present study, serum IL-1β (Th1 cytokine) was positively correlated with serum urea, CK-MB, and LDH in both moderate and severe COVID-19 patients. The other Th1 cytokine, IFN-γ, was also positively correlated with CK-MB in moderate cases and with serum creatinine, urea, CK-MB, and LDH in severe COVID-19 cases. On the other hand, Th2 cytokine, IL-4, was positively correlated with serum urea, CK-MB, and troponin I in moderate COVID-19 patients and with urea, CK-MB, and LDH in severe cases. IL-10, another Th2 cytokine, exhibited significant correlation with CK-MB and troponin I in moderate COVID-19 patients and with serum creatinine, urea, CK-MB, and LDH in severe cases. Thus, it can be concluded that cytokine storm including elevations in Th1 and Th2 cytokines in addition to increases Th17 cytokine (IL-17) can be linked to the impairment effects of COVID-19 on renal and cardiac functions.

Channappanavar and Perlman proved that the CSS can induce apoptosis of the epithelial cells and endothelial cells, and induce leakage from the blood vessels, ultimately causing ARDS and other severe syndromes, as well as mortality [46].

Patients who experience COVID-19 have serious symptoms such as septicemia and ARDS. While most patients have mild symptoms, COVID-19 can also impact the cardiovascular system, triggering myocardial damage, cardiac and endothelial dysfunction, and organ failure. Every so often, kidneys are implicated in both direct and indirect mechanisms. Kidney contribution is always expressed as proteinuria and AKI [47].

This agrees with our clinical evidence, which demonstrated an increase in biomarkers for renal function and cardiac enzymes in both patient groups. Our data demonstrated higher serum creatinine and urea levels in all 100 COVID-19 sufferers. These patients demonstrated a greater frequency of medical complications and intensive care requirements versus patients with normal kidney function. Most patients were diagnosed with AKI during hospital treatment. These results agreed with these of Ahmadian et al. who reported that patients with higher serum creatinine and urea values can develop AKI during hospitalization [47].

European and USA studies demonstrated that COVID-19 causes AKI in 20–40% of ICU inpatients and AKI is considered an adverse prognostic factor and an indication of disease severity [48].

Our data agree with these results as we reported that serum creatinine and urea levels were substantially higher among the severe group versus the healthy control and moderate groups.

Various factors may contribute to the etiology of renal impairment in subjects with COVID-19 [49,50]. The initial influence can be the existence of a cytokine storm that can affect the kidney by inducing septicemia, shock, oxygen depletion, and rhabdomyolysis [51]. The interaction between the heart, kidney, and lung is an additional potential cause of the kidney impairment induced by COVID-19. This agrees with our results in which we revealed elevated CK-MB and LDH activities in both infected groups and elevated troponin I only in the severe group. In COVID-19, the cytokine storm can play a role in AKI through collaborating with kidney resident cells and fostering impairment of tubular and endothelial function. Pro-inflammatory cytokines are able to provoke capillary leak syndrome and the creation of blood clots, which can lead to disseminated intravascular coagulation [52]. Furthermore, cellular death and tissue injuries can occur as a consequence of cytokines circulating in higher levels in the blood. In addition, cytokines can activate macrophages, so erythrocyte phagocytosis and anemia are observed together leading to multiple organ failures [53]. The second mechanism that can explain the COVID-19 renal influence is the binding to ACE-2 receptors that are the main binding domain for SARS-CoV2. Our results showed an elevation in ACE-2 expression levels in both groups; moreover, a significant positive link was observed between serum creatinine and urea and INF-γ and ACE-2 expression levels, particularly in the severe group. Kidney proximal tubules and many other cells can express the ACE-2 receptors [54]. The kidney expresses ACE-2 in the brush border apical membrane of the proximal tubules more than in renal podocytes [55]. Accordingly, it can be postulated that the virus penetrates afferent arterioles, then enters the glomerular capillaries and mainly infects endothelial cells of the glomerulus. Subsequently, the virus infects the podocytes then reaches the tubular fluid, and thereafter binds with its receptors on the proximal tubules [56].

NRP-1, one of the signaling protein families, was proved to function as the gate entry and strengthening factor for the ability of SARS-CoV-2 to infect cells in the laboratory. This cellular surface receptor participates in various facets in SARS-CoV-2 infection, which include the spread into the olfactory bulb from where it can reach the central nervous system and enhance pulmonary expression of NRP-1 in cases of severe COVID-19 [57]. NRP-1 is a receptor that regulates endothelial development and is the focus of anticancer treatments. Nevertheless, its function in all other vascular cells, including pericytes, is unknown [58]. The kidney is a pericyte-rich organ, and pericytes have been linked to several renal pathologies, which include interstitial fibrosis and diabetic nephropathy. Furthermore, severe COVID-19 causes arterial damage, and the up-regulation of NRP-1 plays a crucial role in renal dysfunction [58]. Our results showed overexpression of NRP-1 receptor in severely and moderately infected patients compared with the healthy group. These data agree with those of Gudowska-Sawczuk and Wnuk et al. who noticed that NRP-1 is a crucial receptor that helps SARS-CoV-2 to get to the inside of the host cell and facilitates SARS-CoV-2 spread [57,58,59]. Gudowska-Sawczuk and Mroczko suggested that NRP-1 enables communication between SARS-CoV-2 and ACE-2 [59]. Ahmadian et al. concluded that the pathophysiology of AKI can be linked with COVID-19-specific mechanisms [47]. NRP-1 expression, in the present study, was significantly correlated with serum creatinine and urea levels in severe COVID-19 patients but, in moderate cases, it was significantly correlated with serum urea level only. This led us to suggest that upregulated NRP-1 expression may have a role in deterioration in kidney function due to COVID-19 infection.

Our study demonstrated a link between COVID-19 and cardiac biomarkers in serum. Basu-Ray et al. reported a link between heart failure and COVID-19 [60]. Based upon the reports conducted during the ongoing COVID-19 pandemic, several mechanisms have been proposed for cardiac damage. The direct mechanism implicates viral infiltration in the myocardial tissue, thus leading to cardiomyocyte death and inflammation [61]. The second mechanism is a cytokine storm that takes place in sufferers of severe COVID-19. Several pro-inflammatory cytokines, which include the interleukins (IL-2, IL-10, IL-6, and IL-8), as well as the tumor necrosis factor (TNF)-α, are significantly elevated in severe cases [62]. In this study, a considerable rise in serum LDH, troponin I, CK-MB, IL-1β, IL-4, IL-10, IL-17, and INF-γ levels was recorded in COVID-19 patients compared to healthy controls. These findings agreed with the results of Clerkin et al. who concluded that myocardial injury can be attributed to the accompanying cytokine storm displayed by the raised levels of IL-6, D-dimer, ferritin, LDH, and/or impaired myocardial function from the direct impact of SARS-CoV-2 on the heart [61]. The third mechanism is that the interplay of SARS-CoV-2 with ACE-2 may lead to alterations in the ACE-2 pathways, resulting in an acute pulmonary, cardiac, and endothelial cell injury. Few case reports have demonstrated that SARS-CoV2 can immediately infect the myocardium, triggering viral myocarditis. Nevertheless, in the most cases, myocardial lesions are triggered by the rise in cardiometabolic request linked to systemic infection and continuous oxygen depletion induced by severe pneumonia [63]. This mechanism has been proposed and supports our results—the over expressions of ACE-2 receptors among the two diseased groups versus the healthy group. The fourth mechanism is increased NRP-1, which strongly correlated with the spread of pneumonic SARS-CoV-2 nucleocapsid inside the wall of the left ventricle [64]. This mechanism has been proposed and is supported by our results by the upregulated expressions of NRP-1 among the two COVID-19 groups compared with the healthy group. Our study demonstrated a significant positive correlation between NRP-1 expression and cardiac damage biomarkers (rise of serum LDH and CK-MB activities and troponin I level) in both moderate and severe COVID-19 groups; the correlation was augmented by severity of infection. These findings are in line with those of Mustroph et al. who hypothesized that higher cardiac fibrosis may predispose to progression of severe COVID-19 due to increased cardiac expression of NRP-1, which permits viral cell entry. In addition, troponin I levels are elevated in severe COVID-19 cases [63]. Glinka et al. and Meng et al. concluded that the presence of soluble interleukin-1 receptor-like 1 (sIL1-RL1) in higher levels may provoke expression of NRP-1, the transforming growth factor β1 (TGF-β1) co-receptor [64,65]. Biernacka et al., Kakkar and Lee, and Matilla et al. demonstrated that elevated levels of TGF-β1, either circulating or local, and sIL1-RL1 are a characteristic of cardiac fibrosis [66,67,68], furthermore, enhanced sIL1-RL1-stimulated expression of NRP-1 has been proven in rat cardiomyocytes as well as human cardiac fibroblasts; consequently, it is plausible to validate that NRP-1 levels are high in human fibrotic hearts [69].

## 5. Conclusions

The present study revealed that indicators of renal function (serum creatinine and urea) are positively correlated with IL-10 and INF-γ, in addition to their correlation with ACE-2 and NRP-1 expression levels. Moreover, the important cardiac enzyme, LDH, was positively correlated with all the examined cytokine, NRP-1, and ACE-2 expression levels. Infection with SARS-CoV-2 is associated with abnormalities such as cardiac and renal dysfunctions. Impairment of renal and cardiac function biomarkers in serum might be the result of a cytokine storm and an increase in the expression of ACE-2 and NRP-1 in COVID-19 patients. Early diagnosis of cardiovascular disease and kidney function by clinical examination and laboratory investigations and treatment that is directed towards the reduction of cytokines storm are extremely important. In addition, alterations in the expression of ACE-2 and NRP-1 receptors may play a major role in the associations of cytokine storm and renal and heart dysfunctions due to their facilitation of COVID-19 infection. Thus, understanding the processes and functions of both ACE-2 and NRP-1 can help to lessen the severity of sickness and the danger of death. Clinical trials should be conducted to examine guidance and enhance the management of cardiovascular and kidney aspects of COVID-19.

## Figures and Tables

**Table 1:** Demographic data of studied groups.

	Healthy Controls	Moderate COVID-19 Patients	Severe COVID-19 Patients
**Number of subjects**	50	50	50
**Age**			
**Mean ± SEM**	52.12 ± 1.22	58.24 ± 1.24	57.36 ± 1.43
**Gender number (%)**			
**Male**	25 (50%)	25 (50%)	29 (58%)
**Female**	25 (50%)	25 (50%)	21 (42%)
**BMI (kg/m^2^)**			
**Range**	20.28–28.09	20.8–40.10	20.30–40.10
Mean ± SEM	24.25 ± 0.35	24.73 ± 0.48	25.03 ± 0.52

**Table 2:** Correlations between kidney and heart function biomarkers, cytokine levels and ACE-2 and NRP-1 expression levels in the moderate group.

	Creatinine		Urea		CK-MB		LDH		Troponin I	
	r	*p*	r	*p*	r	*p*	r	*p*	r	*p*
**Creatinine**	1		0.626 ***	0.000	0.069	0.498	0.219 *	0.029	0.140	0.163
**Urea**	0.626 ***	0.000	1		0.360 ***	0.000	0.463 ***	0.000	0.155	0.123
**LDH**	0.219 *	0.029	0.463 ***	0.000	0.165	0.101	1		0.051	0.617
**CK-MB**	0.069	0.498	0.360 ***	0.000	1		0.165	0.101	0.325 **	0.001
**Troponin I**	0.140	0.163	0.155	0.123	0.325 **	0.001	0.051	0.617	1	
**IL1-β**	−0.210 *	0.036	0.258 **	0.010	0.604 ***	0.000	0.292 **	0.003	0.196	0.051
**IL-4**	−0.033	0.747	0.256 *	0.010	0.600 ***	0.000	0.136	0.179	0.346 ***	0.000
**IL-10**	−0.135	0.180	0.090	0.371	0.451 ***	0.000	0.080	0.428	0.215 *	0.031
**IL-17**	−0.157	0.118	0.275 **	0.006	0.628 ***	0.000	0.298 **	0.003	0.207 *	0.039
**IFN-γ**	−0.130	0.196	0.174	0.083	0.520 ***	0.000	0.176	0.080	0.124	0.221
**NRP-1**	−0.018	0.860	0.334 **	0.001	0.653 ***	0.000	0.198 *	0.048	0.261 **	0.009
**ACE-2**	−0.113	0.262	0.193	0.054	0.543 ***	0.000	0.023	0.823	0.260 **	0.009

*, **, and *** correlation was significant at *p* < 0.05, *p* < 0.01, and *p* < 0.001, respectively.

**Table 3:** Correlations between kidney function biomarkers, cardiac function bioindicators and cytokine levels and ACE-2 and NRP-1 expression levels in the severe group.

	Creatinine		Urea		CK-MB		LDH		Troponin I	
	r	*p*	r	*p*	r	*p*	r	*p*	r	*p*
**Creatinine**	1		0.843 ***	0.000	0.230 *	0.022	0.027	0.792	−0.052	0.611
**Urea**	0.843 ***	0.000	1		0.395 ***	0.000	0.197 *	0.049	−0.003	0.973
**LDH**	0.027	0.792	0.197 *	0.049	0.409 ***	0.000	1		0.399 ***	0.000
**CK-MB**	0.230 *	0.022	0.395 ***	0.000	1		0.409 ***	0.000	0.310 **	0.002
**Troponin I**	−0.052	0.611	−0.003	0.973	0.310 **	0.002	0.399 ***	0.000	1	
**IL1-β**	0.158	0.117	0.445 ***	0.000	0.601 ***	0.000	0.353 ***	0.000	0.167	0.098
**IL-4**	0.077	0.446	0.223 *	0.026	0.397 ***	0.000	0.319 **	0.001	0.201 *	0.045
**IL-10**	0.228 *	0.023	0.451 ***	0.000	0.690 ***	0.000	0.467 ***	0.000	0.175	0.081
**IL-17**	0.181	0.072	0.335 **	0.001	0.682 ***	0.000	0.507 ***	0.000	0.383 ***	0.000
**IFN-γ**	0.213 *	0.033	0.423 ***	0.000	0.545 ***	0.000	0.407 ***	0.000	0.088	0.386
**NRP-1**	0.300 **	0.002	0.443 ***	0.000	0.624 ***	0.000	0.328 **	0.001	0.301 **	0.002
**ACE-2**	0.204 *	0.041	0.438 ***	0.000	0.586 ***	0.000	0.557 ***	0.000	0.147	0.144

*, **, and *** correlation was significant at *p* < 0.05, *p* < 0.01, and *p* < 0.001, respectively.

## Data Availability

The data are contained within the article.

## Abbreviations

ACE-2: angiotensin-converting enzyme-2; AKI: acute kidney injury; ARDS: acute respiratory distress syndrome; BMI: body mass index; CKD: chronic kidney disease; CHD: coronary heart disease; COVID-19: coronavirus disease 2019; CSS: cytokine storm syndrome; HF: heart failure; ICU: intensive care unit; IL: interleukin; IFN-γ: interferon-gamma; mRNA: messenger ribonucleic acid; NRP-1: neuropilin-1; qRT-PCR: quantitative real-time polymerase chain reaction; RAS: renin–angiotensin system; TGF-β: transforming growth factor-beta; Th: T-helper; TNFα: tumor necrosis factor-α; WHO: World Health Organization.
